# Crystallographic data of an importin-α3 dimer in which the two protomers are bridged by a bipartite nuclear localization signal

**DOI:** 10.1016/j.dib.2023.108988

**Published:** 2023-02-16

**Authors:** Yoshiyuki Matsuura

**Affiliations:** a Department of Pharmaceutical Sciences, School of Pharmacy, International University of Health and Welfare, Tochigi 324-8501, Japan; b Division of Biological Science, Graduate School of Science, Nagoya University, Nagoya 464-8602, Japan

**Keywords:** Nuclear import, Nuclear transport receptor, Nuclear localization signal, Crystallization, Dimerization

## Abstract

53BP1 (TP53-binding protein 1), a key player in DNA double-strand break repair, has a classical bipartite nuclear localization signal (NLS) of sequence 1666-GKRKLITSEEERSPAKRGRKS-1686 that binds to importin-α, a nuclear import adaptor protein. Nucleoporin Nup153 is involved in nuclear import of 53BP1, and the binding of Nup153 to importin-α has been proposed to promote efficient import of classical NLS-containing proteins. Here, the ARM-repeat domain of human importin-α3 bound to 53BP1 NLS was crystallized in the presence of a synthetic peptide corresponding to the extreme C-terminus of Nup153 (sequence: 1459-GTSFSGRKIKTAVRRRK-1475). The crystal belonged to space group *I*2, with unit-cell parameters *a* = 95.70, *b* = 79.60, *c* = 117.44 Å, *β* = 95.57°. The crystal diffracted X-rays to 1.9 Å resolution, and the structure was solved by molecular replacement. The asymmetric unit contained two molecules of importin-α3 and two molecules of 53BP1 NLS. Although no convincing density was observed for the Nup153 peptide, the electron density corresponding to 53BP1 NLS was unambiguous and continuous along the entire length of the bipartite NLS. The structure revealed a novel dimer of importin-α3, in which two protomers of importin-α3 are bridged by the bipartite NLS of 53BP1. In this structure, the upstream basic cluster of the NLS is bound to the minor NLS-binding site of one protomer of importin-α3, whereas the downstream basic cluster of the same chain of NLS is bound to the major NLS-binding site of another protomer of importin-α3. This quaternary structure is distinctly different from the previously determined crystal structure of mouse importin-α1 bound to the 53BP1 NLS. The atomic coordinates and structure factors have been deposited in the Protein Data Bank (accession code 8HKW).

**Specifications Table**

**Table:** 

Subject	Biology
Specific subject area	Structural biology
Type of data	Table, figure, structure coordinates, structure factors
How the data were acquired	X-ray diffraction dataset was collected to 1.9 Å resolution at the Photon Factory beamline BL-17A. X-ray wavelength: 0.98 Å Detector type: EIGER X 16 M Software used for data integration: iMosflm
Data format	Raw and analyzed
Description of data collection	Proteins were expressed from bacterial host. Complex of proteins were purified and crystallized. Diffraction data from a single crystal collected at synchrotron radiation source
Data source location	Institution: International University of Health and Welfare City/Town/Region: Tochigi Country: Japan
Data accessibility	The data (coordinates and structure factors) [1] reported in this article have been deposited in the protein data bank (PDB) with accession code 8HKW. Repository name: Protein Data Bank (PDB) Data identification number: 10.2210/pdb8hkw/pdb. Direct URL to data: http://doi.org/10.2210/pdb8hkw/pdb

## Value of the Data

• The significance of importin-α oligomerization has been debated in nuclear transport field. The data reported here extends our knowledge on importin-α oligomerization and will benefit researchers studying the structure and function of nuclear transport receptors.
• The purification procedure, crystallization condition, and a photograph of the crystal used in this work serve as a useful reference for researchers interested in structural study of importin-α-cargo complexes.
• Although the quaternary structure reported here could be an artifact of the crystallization process, the data enriches structural database of what can happen during crystallization of protein-peptide complexes, and will benefit researchers interested in protein crystallization.
• The molecular mechanism of interactions between nucleoporins and nuclear transport receptors is not fully understood, and remains an important issue in nuclear transport field. The data that convincing electron density corresponding to Nup153 was not observed in the crystal structure reported here is a negative data useful for researchers studying nucleoporin-importin interactions.

## 1 Objective

The initial objective of this work was to gain structural insights into how Nup153 interacts with importin-α and promotes nuclear import of NLS-containing proteins such as 53BP1. However, this work serendipitously led to a discovery of a novel quaternary structure of importin-α-NLS complexes that can be formed at least in the crystal.

## 2 Data Description

X-ray diffraction dataset up to 1.9 Å resolution was collected from a single crystal of the ARM-repeat domain of human importin-α3 bound to the bipartite NLS of 53BP1 (human 53BP1 residues 1665–1686; [2]), grown in the presence of a Nup153 peptide (referred to as Nup153C), at the Photon Factory beamline BL-17A. The Nup153C peptide corresponds to the extreme C-terminus of human Nup153 (residues 1459–1475). Previous studies have shown that Nup153 is involved in nuclear import of 53BP1 [3] and that the interaction between Nup153 and importin-α is required for efficient nuclear import of classical NLS-containing proteins [4]. It has been proposed that the extreme C-terminus of Nup153 binds directly to importin-α [4,5].

**Fig. 1:**
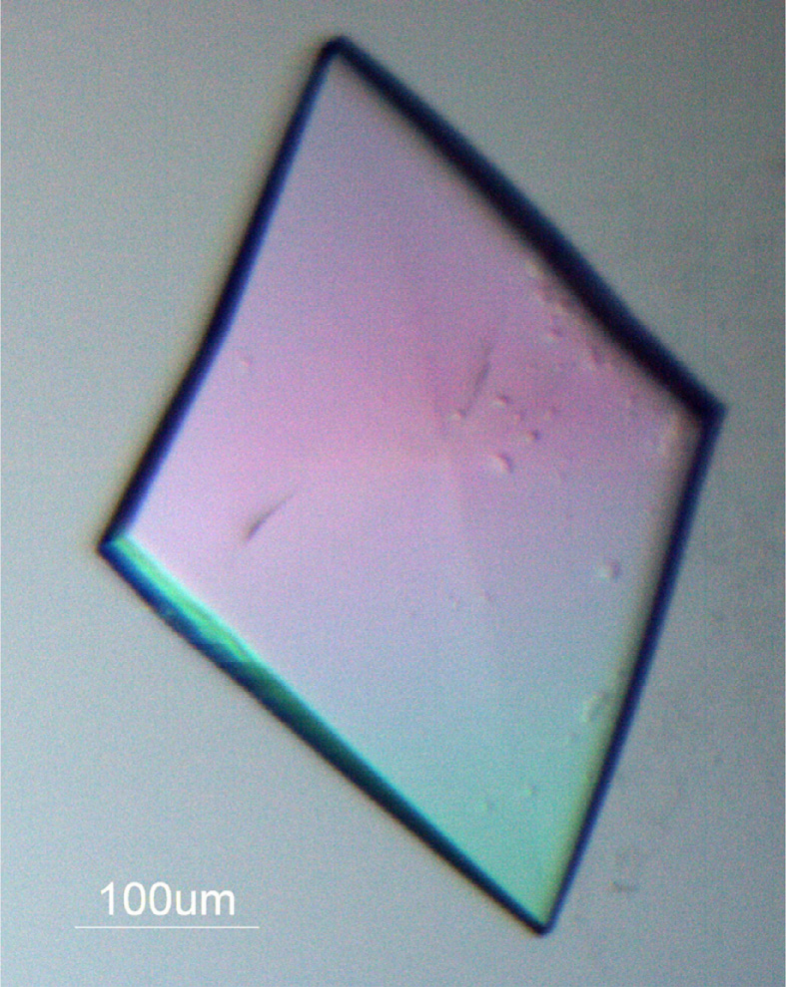
A crystal of the complex between the ARM-repeat domain of importin-α3 and the 53BP1 NLS grown in the presence of the Nup153C peptide. Scale bar, 100 μm.

**Fig. 2:**
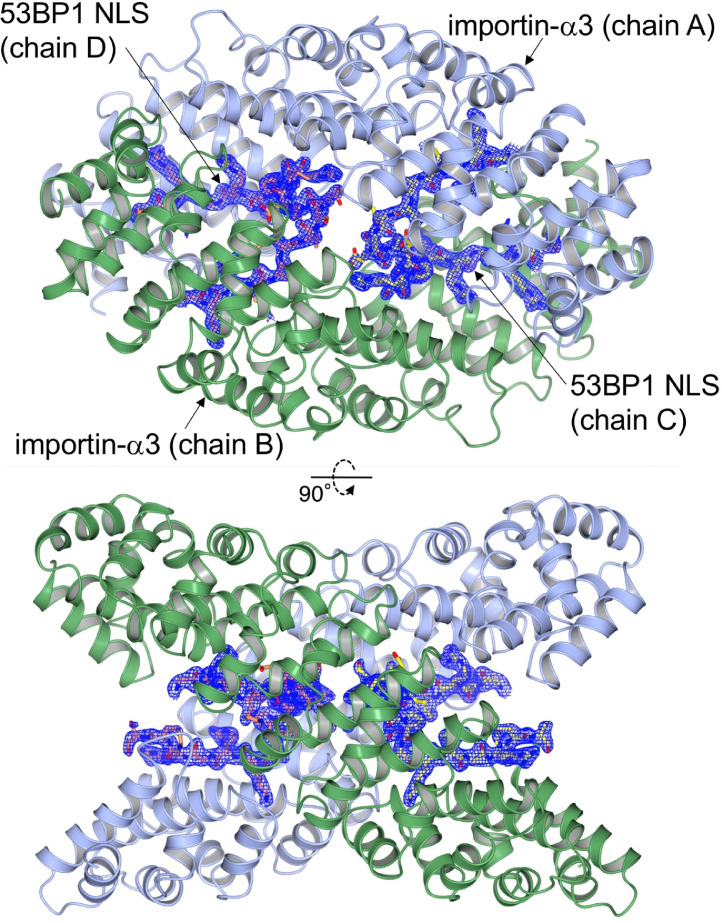
The overall structure of the ARM-repeat domain of importin-α3 (ribbon representation, chains A and B) in complex with the 53BP1 NLS (stick representation, chains C and D). All polypeptide chains in the asymmetric unit are shown in two orthogonal views. The 2*F*_O_−*F*_C_ electron density map covering the 53BP1 NLS (contoured at 1.0σ) is shown in blue mesh.

Fig. 1 shows the plate-shaped crystal used for data collection. The structure was solved by molecular replacement, with the structure of importin-α3 bound to Hendra virus W protein C-terminus (PDB code, 6BW9) [6] as a search model. The structure was refined to free and working *R*-factor values of 22.14% and 20.06%, respectively. Fig. 2 shows the overall structure of the asymmetric unit, which contained two molecules of importin-α3 and two molecules of the 53BP1 NLS. Data collection and refinement statistics are shown in Table 1.

**Table 1:** Crystallographic statistics.

Data collection	
Space group	*I*2
Unit cell dimensions	
*a, b, c* (Å)	95.70, 79.60, 117.44
*α, β, γ* (degree)	90.00, 95.57, 90.00
Wavelength (Å)	0.98
X-ray source	Photon Factory BL-17A
Resolution range (Å)^a^	27.34–1.90 (1.94–1.90)
No. of measured reflections^a^	203,952 (13,341)
No. of unique reflections^a^	68,772 (4433)
Completeness (%)^a^	99.4 (99.7)
*R*_p.i.m._ (%)^a^	6.5 (82.8)
Mean *I*/σ(*I*)^a^	4.9 (0.7)
Mean *I* half-set correlation CC(1/2)^a^	0.993 (0.338)
Multiplicity^a^	3.0 (3.0)
Wilson *B*-factor (Å^2^)	25.9
**Refinement**	
Resolution range (Å)^a^	27.34–1.90 (1.93–1.90)
*R*_work_ (%)^a^	20.06 (37.87)
*R*_free_ (%)^a^	22.14 (40.37)
No. of atoms	
Protein	6768
Water	502
No. of amino acids	872
Mean *B*-factor (Å^2^)	
importin-α3	44.6
53BP1	51.5
Water	46.1
RMSD from ideality	
Bond lengths (Å)	0.003
Bond angles (degree)	0.535
Protein geometry^b^	
Rotamer outliers (%)	0.91
Ramachandran favored (%)	98.84
Ramachandran outliers (%)	0
Cβ deviations > 0.25 Å (%)	0

a Values in parentheses are for the highest-resolution shell.

b MolProbity was used to analyze the structure.

As shown in Fig. 2, the 53BP1 NLS was unambiguously identified in the electron density map, and the density was continuous along the entire length of this bipartite NLS. However, no convincing density was observed for the Nup153C peptide. This quaternary structure of importin-α3–53BP1 NLS complex is distinctly different from the previously reported crystal structure of mouse importin-α1–53BP1 NLS complex, in which one molecule of 53BP1 NLS is bound along the concave surface of one molecule of importin-α1 [7].

## 3 Experimental Design, Materials and Methods

### 3.1 Protein expression and purification for crystallization

To prepare the ARM-repeat domain of importin-α3 in complex with the 53BP1 NLS, untagged importin-α3 (human, residues 70–485) and glutathione *S*-transferase (GST)-tagged 53BP1 NLS (human, residues 1665–1686) were co-expressed in the *E. coli* host strain BL21-CodonPlus(DE3)RIL (Stratagene) at 20°C from pRSFDuet-1 (Novagen) and pGEX-TEV [8], respectively. The cells were harvested, frozen in liquid nitrogen, and stored at −25°C. The cell pellets were resuspended in buffer A [10 mM Tris–HCl pH 7.5, 150 mM NaCl, 1 mM EDTA, 1 mM phenylmethylsulfonyl fluoride (PMSF), and 7 mM 2-mercaptoethanol] and lysed by sonication on ice. All subsequent steps were performed at 4°C. Tween20 was added to 0.1%, and clarified lysates were incubated with glutathione-Sepharose 4B (GE Healthcare) for 4 h. After washing the beads with buffer B (10 mM Tris–HCl pH 7.5, 150 mM NaCl, 0.05% Tween20, and 2 mM 2-mercaptoethanol), the GST-tag was removed with His-TEV protease (60 μg/ml) overnight. The importin-α3–53BP1 NLS complex released from the resin was finally purified over Superdex200 (GE Healthcare) in buffer C (10 mM Tris–HCl pH 7.5, 150 mM NaCl, and 2 mM 2-mercaptoethanol). The complex was concentrated to 0.6 mM using a 3 kDa molecular weight cutoff Amicon Ultra centrifugal filter (Millipore).

### 3.2 Crystallization, data collection, and structure determination

A synthetic peptide (referred to as Nup153C) corresponding to the extreme C-terminus (residues 1459–1475) of human Nup153 (^1459^GTSFSGRKIKTAVRRRK^1475^) was synthesized by GenScript. A plate-shaped crystal was obtained using hanging drop vapor diffusion at 20 °C with 1.5 μl of a protein solution (0.58 mM importin-α3–53BP1 NLS complex and 0.71 mM Nup153C in buffer C) mixed with 1.5 μl of mother liquor (0.2 M LiNO_3_ and 25% PEG3350) over a reservoir of 600 μl of mother liquor. The crystal was briefly soaked in a cryoprotection solution (0.2 M LiNO_3_, 28% PEG3350, 6% PEG400, and 0.5 mM Nup153C) for less than 30 s, and flash-cooled in liquid nitrogen. X-ray diffraction datasets were collected at 95 K at the Photon Factory beamline BL-17A using an EIGER X 16 M detector at a wavelength of 0.98 Å. Diffraction data were processed using iMOSFLM and CCP4 programs [9]. The structure was solved by molecular replacement using MOLREP [10], with the structure of importin-α3 bound to Hendra virus W protein C-terminus (PDB code, 6BW9) [6] as a search model. The atomic model was iteratively rebuilt using COOT [11] and refined using PHENIX [12]. MolProbity [13] was used to validate the final model. CCP4MG [14] was used to generate structural figures. The atomic coordinates and structure factors have been deposited in the PDB with accession code 8HKW [1].

## Ethics Statements

This work meets the ethical requirements for publication in this journal. This work does not involve human subjects, animal experiments, or any data collected from social media.

## CRediT Author Statement

**Yoshiyuki Matsuura:** Conceptualization, Investigation, Writing – Original Draft, Funding acquisition.

## CRediT authorship contribution statement

**Yoshiyuki Matsuura:** Conceptualization, Investigation, Writing – original draft, Funding acquisition.

## Declaration of Competing Interest

The authors declare that they have no known competing financial interests or personal relationships that could have appeared to influence the work reported in this paper. The authors declare the following financial interests/personal relationships which may be considered as potential competing interests:

## Data Availability

Crystal structure of importin-alpha3 bound to the 53BP1 nuclear localization signal (Original data) (Protein Data Bank).
